# Somatic and germline expression of *piwi *during development and regeneration in the marine polychaete annelid *Capitella teleta*

**DOI:** 10.1186/2041-9139-2-10

**Published:** 2011-05-05

**Authors:** Vincent C Giani, Emi Yamaguchi, Michael J Boyle, Elaine C Seaver

**Affiliations:** 1 Kewalo Marine Laboratory, PBRC/University of Hawaii, 41 Ahui St., Honolulu, HI 96813, USA; 2 Smithsonian Marine Station, 701 Seaway Drive, Fort Pierce, FL 34949, USA

## Abstract

**Background:**

Stem cells have a critical role during adult growth and regeneration. Germline stem cells are specialized stem cells that produce gametes during sexual reproduction. *Capitella teleta *(formerly *Capitella *sp. I) is a polychaete annelid that reproduces sexually, exhibits adult growth and regeneration, and thus, is a good model to study the relationship between somatic and germline stem cells.

**Results:**

We characterize expression of the two *C. teleta *orthologs of *piwi*, genes with roles in germline development in diverse organisms. *Ct-piwi1 *and *Ct-piwi2 *are expressed throughout the life cycle in a dynamic pattern that includes both somatic and germline cells, and show nearly identical expression patterns at all stages examined. Both genes are broadly expressed during embryonic and larval development, gradually becoming restricted to putative primordial germ cells (PGCs) and the posterior growth zone. In juveniles, *Ct-piwi1 *is expressed in the presumptive gonads, and in reproductive adults, it is detected in gonads and the posterior growth zone. In addition, *Ct-piwi1 *is expressed in a population of putative PGCs that persist in sexually mature adults, likely in a stem cell niche. *Ct-piwi1 *is expressed in regenerating tissue, and once segments differentiate, it becomes most prominent in the posterior growth zone and immature oocytes in regenerating ovaries of regenerating segments.

**Conclusions:**

In *C. teleta, piwi *genes may have retained an ancestral role as genetic regulators of both somatic and germline stem cells. It is likely that *piwi *genes, and associated stem cell co-regulators, became restricted to the germline in some taxa during the course of evolution.

## Background

Stem cells are essential for animal development and adult tissue homeostasis, and they can presumably differentiate into many specialized cell types. Specialized stem cells called primordial germ cells (PGCs) are populations of undifferentiated stem cells in sexually reproducing animals that will exclusively give rise to the germ cells, either spermatocytes or oocytes [1]. These germline stem cells insure that genetic information is passed to the next generation. In some animals, germline stem cells are segregated from somatic cells during embryonic development. Two distinct mechanisms of germline specification have been described: preformation and epigenesis [2]. According to the preformationist mode, germ cells are specified by maternally inherited determinants present within the egg. In the case of epigenesis, germ cells are not specified until later in development, and arise as a result of inductive signals from surrounding tissues. In some basally branching animals, there is not such a separation between the germline and the soma in the embryo, and germ cells can be segregated from somatic cells throughout the life cycle. This raises the question of the relationship between somatic stem cells and germline stem cells. It has been proposed that germline stem cells arose from a preexisting multipotent progenitor lineage that later in evolution became a restricted sublineage [3]. If this is the case, have some bilaterian animals retained an ancestral association between germline stem cells and somatic stem cells? Are core regulatory genes shared between multipotent stem cells and germline stem cells in some animal groups?

Studies in annelids are likely to provide insights into the relationship between somatic and germline stem cells. Polychaete annelids are highly variable in their reproductive patterns and many species can regenerate their heads, tails or both [4]. The polychaete annelid *Capitella teleta*, formerly known as *Capitella *sp. I [5] is a simple-bodied, marine polychaete annelid that undergoes sexual reproduction, continuously generates segments during its lifetime, and exhibits robust posterior regeneration, including regeneration of its ovaries. In *C. teleta*, there are males, females and hermaphrodites; males can transform into hermaphrodites as a result of changing environmental conditions [6]. Gametogenesis and the location of the reproductive organs in *C. teleta *have previously been described in detail [5,7,8]. The testes are specialized regions of the lateral peritoneum in the seventh and eighth segments and lack a well developed anatomical structure. Several later stages of spermatogenesis occur within the coelomic cavity, and in mature males, sperm are stored in paired genital ducts (coelomoducts) at the boundary between segments 7 and 8. The genital ducts are trumpet-shaped structures that open into the ventro-lateral coelomic cavity on one end and on the other end have a narrow canal that terminates in an intersegmental pore, separate from metanephridia present in the same segment. Females have well-defined segmentally repeated ovaries present in 10 to 12 continuous segments beginning with the first abdominal segment. The ovaries are ventrally positioned, paired structures adjacent to the gut tube. Each ovary is suspended by mesenteries on the ventral side of the coelomic cavity and is macroscopically visible. The sac of the ovary is composed of follicle cells that are formed by modified coelomic peritoneal cells. Vitellogenesis (and potentially a proliferative phase) occurs within the ovaries, and each ovary contains 5 to 20 oocytes at multiple stages of oogenesis, including mature oocytes. Distinct stages are spatially segregated within the ovary, with proliferative oogonia and pre-vitellogenic oocytes in the medial region of the ovary and mature oocytes localized to the lateral region [7]. Individuals of both sexes can reproduce multiple times. Hermaphroditic animals display both male and female characteristics. The detailed knowledge of gametogenesis and adult anatomy, generation of segments as an adult, available fate map [9], and the ability to regenerate make *C. teleta *a good lophotrochozoan model to study the segregation of the germline and the relationship between somatic and germline stem cells.

In contrast to the detailed knowledge of gametogenesis for many polychaetes, far less is known about primordial germ cells and the origin of the germline in polychaetes [10,11]. Along with the *vasa *and *nanos *genes, *piwi *has essential roles during germline development. *Piwi *genes are members of the Argonaute (Ago) family of proteins, which function through their interactions with small RNAs (sRNAs). In *Drosophila melanogaster *and mammals, piwi proteins bind to special classes of sRNA molecules such as repeat-associated small interacting RNAs (rasiRNAs), and piwi-interacting RNAs (piRNAs) [12-14]. PiRNAs are often complementary to transposon sequences and can silence transposable elements in the germline [15]. *Piwi *genes can thus affect germline determination, germline maintenance, gametogenesis, stem cell self-renewal, RNA interference (RNAi), and transposon silencing [14]. In *D. melanogaster*, *piwi *is expressed in embryos as well as adult gonads [12]. Within ovaries, *piwi *is expressed in both somatic and germline cells [16]. Mutations within the *piwi *gene of *D. melanogaster *cause male sterility due to defects in spermatogenesis, and mutant females are deficient in germline cells [17]. In mice, *miwi *(mouse homolog of *piwi*) is normally expressed during spermatogenesis where it is largely restricted to the testis [14], and *miwi *knockout mice exhibit male sterility, characterized by a block at the early spermatid stage [14]. *Piwi *family genes are also expressed in germ cells across a broad range of taxa, including zebrafish [18], sea urchins [19], ctenophores [20]), and jellyfish [21], although the function and molecular mechanism of its action is only known for a few species. There are limited examples of expression studies for *piwi *genes in annelids. These include characterization of a *piwi *gene ortholog in the gonads and during regeneration in the oligochaete annelid worm *Enchytraeus japonensis *[22], and expression in the PGCs of larvae and juveniles in the polychaete annelid *Platynereis dumerilii *[23].

In this study, we present a comprehensive expression analysis for the two *piwi *paralogs in the genome of the polychaete annelid *C. teleta*. Previously, Dill and Seaver [24] reported that orthologs of *vasa *and *nanos *are expressed in both the germline and somatic cells, primarily in cells of the posterior growth zone of the adult. If these germline regulators as well as *piwi *also function in somatic stem cells, one might predict they are expressed in regenerating tissue. We examined *piwi *expression during embryonic and larval development, and during adult growth and regeneration. Both *Ct-piwi1 *and *Ct-piwi2 *are expressed throughout the life cycle of *C. teleta*, from early cleavage stage embryos to reproductive adult worms and during gametogenesis. In addition, *Ct-piwi1 *has a complex and dynamic expression pattern during posterior regeneration in both somatic and germline precursors.

## Results

### Phylogenetic analyses of *C. teleta *Piwi

**Figure 1:**
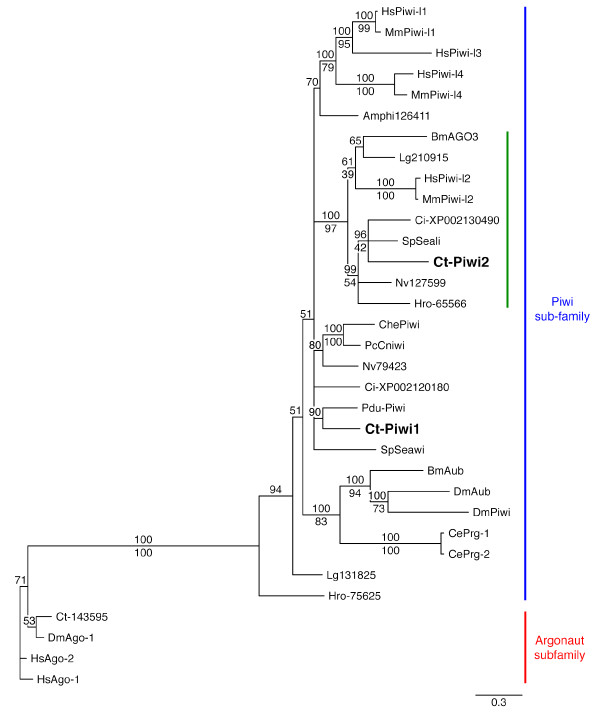
**Phylogenetic analysis of *Ct-Piwi1 *and *Ct-Piwi2 *sequences**. The argonaute and piwi subfamilies are indicated by a red and blue line, respectively. *Ct-Piwi1 *and *Ct-Piwi2 *are both members of the Piwi subfamily of Argonaute proteins. *Ct-Piwi2 *clusters in a sub-group, indicated by a green line, containing other Piwi2 or Piwi-like proteins from broad metazoan taxa. The tree shown is a Bayesian consensus tree with posterior probabilities placed above the nodes. Maximum likelihood bootstrap support values are included where the tree topology agreed with the Bayesian analysis, and are placed below the nodes. Species abbreviations: Amphi, *Branchiostoma floridae*; Bm, *Bombyx mori*; Ce, *Caenorhabditis elegans*; Ct-, *Capitella teleta*; Ci-, *Ciona intestinalis*; Che, *Clytia hemisphaerica*; Dm, *Drosophila melanogaster*; Hro-, *Helobdella robusta*; Hs, *Homo sapiens*; Lg, *Lottia gigantea*; Mm, *Mus musculus*; Nv, *Nematostella vectensis*; Pdu-, *Platynereis dumerilii*; Pc, *Podocoryne carnea*; Sp, *Strongylocentrotus purpuratus*. JGI protein ID number is indicated for *C. teleta *outgroup and sequences from *H. robusta*, *L. gigantea*, and *N. vectensis*. NCBI accession number is indicated for *C. intestinalis*. Gene locus number is indicated for *B. floridae*.

Searches of the *C. teleta *genome identified two putative *piwi *homologs, which we call *Ct-piwi1 *and *Ct-piwi2*. Both predicted open reading frames contain conserved PAZ and PIWI domains characteristic of *piwi *genes. The PIWI domain in both Ct-Piwi1 and Ct-Piwi2 is located near the 3' end of the ORF, which is typical of *piwi *genes [25]. Phylogenetic analyses were conducted by Bayesian and maximum likelihood methods. Both Ct-Piwi1 and Ct-Piwi2 cluster within the Piwi subfamily of the Ago family of proteins, separately from the Argonaute subfamily (Figure 1). There is 100% Bayesian posterior probability support for the Piwi subfamily node, as well as 100% maximum likelihood bootstrap support. *Ct-Piwi1 *and *Ct-Piwi2 *are more closely related to Piwi paralogs from other animals than to each other; we infer that these two genes do not represent a recent gene duplication. Indeed, most of the animals included in these analyses have at least two paralogs that are divergent from each other, which suggests a deep root for this duplication, perhaps in the bilaterian or even metazoan ancestor. While there is some lack of resolution among the Piwi1, Piwi-like1, Piwi-like3, and Piwi-like4 proteins, the Piwi2 and Piwi-like2 sequences group closely together, with 100% Bayesian posterior probability support and 97% maximum likelihood bootstrap support for this node. Ct-Piwi2 belongs to this Piwi2 sub-group, which includes representatives from a broad range of metazoan taxa.

### Regenerative capabilities of *C. teleta*

**Figure 2:**
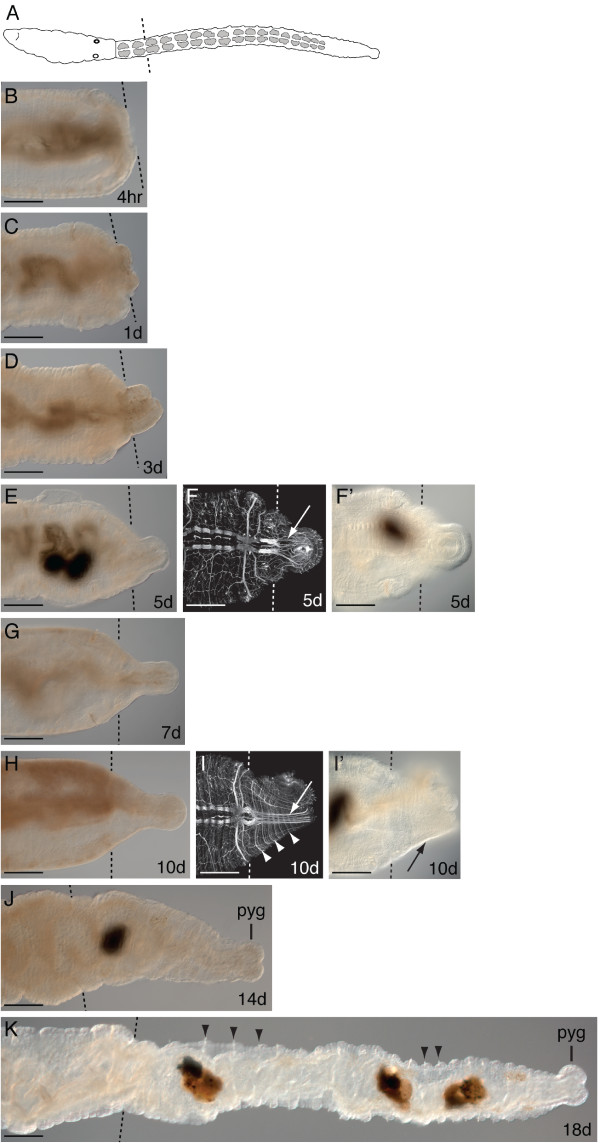
**Time course of posterior regeneration in *C. teleta *from 4 hours to 18 days post-amputation**. Adults were amputated at the 12^th ^segment. Panels shown are posterior ends of amputated adults. All animals are oriented in a ventral view with anterior to the left. Black and white dotted lines indicate cut site in all panels. Dark shapes in **E**, **F'**, **I'**, **J **and **K **are within the lumen of the gut. Images **F **and **I **are Z-stack projections of confocal micrographs of the posterior end of a regenerate labeled with anti-acyetylated tubulin. Abbreviation: pyg, pygidium. (**A**) Schematic of an adult worm indicating the cut site at the 12^th ^segment. (**B**) Adult worm four hours post-amputation. (**C**) one day post-amputation. (**D**) three days post-amputation. (**E**) five days post-amputation. (**F**) Confocal micrograph of a worm five days post-amputation showing longitudinal axons extending into the regenerating tissue (white arrow). **(F') **Corresponding DIC image of the same worm shown in F. **(G) **seven days post-amputation. **(H) **10 days post-amputation. **(I) **Confocal micrograph of a worm 10 days post-amputation showing longitudinal axons (white arrow) and circular nerves (white arrowheads) in the regenerating tissue. **(I') **Corresponding DIC image of the same adult worm in I; note the lack of external segmentation (black arrow). **(J) **14 days post-amputation worm with a distinct pygidium. **(K) **Adult 18 days post-amputation with numerous differentiated segments in the regenerate. Black arrowheads point to chaetae in two sets of adjacent segments. Scale bar, 50 μm for all panels.

*C. teleta *has the ability to regenerate lost tissue, and upon amputation will regenerate posterior segments [26]. To aid our interpretations of *piwi *expression, we characterized posterior regeneration in reproductive adults (eight weeks post-metamorphosis). In *C. teleta*, there are two distinct body regions: segments 1 to 9 are the thoracic segments, and 40 to 50 abdominal segments are continuously added posteriorly throughout adult development. Transverse amputations were made on adult male and female worms at the segment boundary between the 11^th ^and 12^th ^segment (Figure 2A, dotted line). The rate of regeneration varies among individuals; this variation becomes more pronounced after five days post-amputation, and is likely due to environmental conditions. Within four hours of amputation, wound healing occurs by contraction of the severed edges of the body wall (Figure 2B). The gut is closed off during early stages of regeneration by formation of an intact epithelium covering the wound. At one day post-amputation, the wound has fully healed and a small blastema (mass of undifferentiated cells) is visible (Figure 2C). Between one and three days post-amputation, the blastema grows bigger. In addition, the anus has reopened and the worm can feed and excrete ingested material (Figure 2D). Between three and seven days post-amputation, the blastema continues to grow and elongates, but there are no external signs of segmentation (Figure 2E-G). At five days post-amputation, axons can be observed extending from the severed longitudinal nerves into the blastema, likely invading the regenerating tissue from cell bodies in the pre-existing tissue (Figure 2F). The blastema has a smaller diameter relative to the pre-existing tissue, and a distinct pygidium and posterior growth zone appear between 10 and 14 days post-amputation (Figure 2J). Typically, several small segments also become morphologically apparent between 10 and 14 days post-amputation (Figure 2H-J). Nascent segments are initially visible by the appearance of forming ganglia and circular peripheral nerves extending from the ventral nerve cord (Figure 2I, I', arrow, arrowheads); at this stage there are not yet external signs of segmentation (Figure 2I', arrow). The formation of chaetae and intersegmental furrows of the ectoderm occur a few days later (Figure 2K, arrowheads). When segments form, multiple small segments appear rather than a single segment at a time. As many as 20 segments have regenerated by 18 days post-amputation (Figure 2K).

### *Ct-piwi1 *and *Ct-piwi2 *embryonic and larval expression patterns

**Figure 3:**
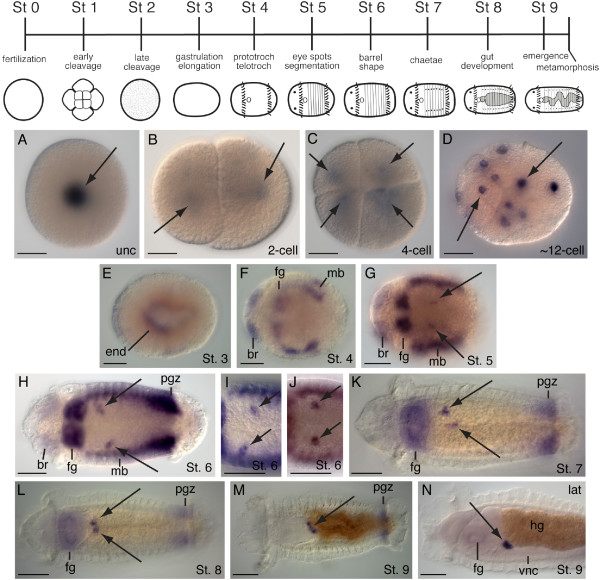
***Ct-piwi1 *expression patterns during embryonic and larval development**. Except for cleavage stages (**A-D**), all animals are ventral views with anterior to the left. **N **is a lateral view with ventral down and anterior to the left. Stages shown are indicated in the lower-right of each panel. At the top is a developmental staging chart for *C. teleta*. Abbreviations: br, brain; end, endoderm; fg, foregut; hg, hindgut; mb, mesodermal bands; pgz; posterior growth zone; vnc, ventral nerve cord. (**A**) Expression in the cytoplasm of uncleaved zygote (arrow). (**B**) Expression is in both cells of a 2-cell stage embryo (arrows). (**C**) Animal view of a four-cell stage embryo showing expression in every cell (arrows). (**D**) Expression throughout the embryo (arrows). (**E**) Endodermal expression in a stage 3 larva. (**F**) Expression in the brain, foregut, and mesodermal bands. (**G**) *Ct-piwi1 *is expressed in two cell clusters on the ventral side of the trunk (black arrows), and in the brain, foregut, and mesodermal bands. (**H**) Expression in the brain, foregut, mesodermal bands, posterior growth zone and in two asymmetrically positioned ventro-lateral cell clusters (arrows). The left cluster is more anterior. (**I**) Two asymmetrically positioned ventro-lateral cell clusters with the right cluster more anterior. (**J**) Two bilaterally symmetric ventro-lateral cell clusters. (**K**) The two *Ct-piwi-*expressing cell clusters are located close to the midline (arrows). Expression persists in the foregut and posterior growth zone. (**L**) The two *Ct-piwi1-*expressing cell clusters are in close proximity near the ventral midline (arrows). Expression persists in the foregut and posterior growth zone. (**M**) By stage 9, expression is limited to a single cluster at the ventral midline (arrow) and posterior growth zone. (**N**) Lateral view showing expression in a single cluster in between the gut and ventral nerve cord at the foregut/hindgut boundary. *Scale bar*, 50 μm for all panels.

We characterized expression of the two *C. teleta piwi *genes during embryonic and larval development by whole mount *in situ *hybridization. *C. teleta *development has been previously described and follows an established staging system (Figure 3, top) [27]. In uncleaved zygotes, two-cell and four-cell stage embryos, *Ct-piwi1 *transcripts can only be detected after an extended color reaction (Figure 3A-C, arrows). In subsequent cleavage stages, *Ct-piwi1 *is broadly expressed in most if not all cells (Figure 3D, arrows). Gastrulation occurs during stage 3, and at this stage the *Ct-piwi1 *expression pattern becomes more restricted within the embryo. Near the end of gastrulation and following closure of the blastopore, *Ct-piwi1 *is transiently expressed in the endoderm (Figure 3E). In early stage larvae (stage 4), *Ct-piwi1 *is expressed in several discrete domains including the presumptive brain, foregut, and mesodermal bands (Figure 3F). These expression domains persist into stage 5, and in addition, two small ventro-lateral clusters of cells appear in the mid-body segments. These clusters are medial to the mesodermal bands (Figure 3G, arrows), and become more easily detected at later larval stages. At stage 6, brain expression weakens, while expression becomes more apparent in the foregut, trunk mesoderm, and a band of *Ct-piwi1*-expressing cells immediately anterior to the telotroch that corresponds to the posterior growth zone (Figure 3H). In addition, expression in the two ventro-lateral cell clusters becomes more prominent; each cluster contains two to five cells and is positioned within the mesoderm (Figure 3H, arrows). There is variation in the position of the ventro-lateral cell clusters within segments 4 and 5 in stage 6 larvae, and three distinct patterns are observed: (1) asymmetrically positioned clusters with the left cluster more anterior (n = 14/35) (Figure 3H, arrows), (2) asymmetrically positioned clusters with the right cluster more anterior (n = 11/35) (Figure 3I, arrows), and (3) bilaterally symmetric cell clusters (n = 10/35) (Figure 3J, arrows). By stages 7 and 8, expression is limited to the foregut, posterior growth zone, and ventro-lateral cell clusters (Figure 3K and Figure 3L, arrows). At these stages, the position of the ventro-lateral cell clusters is closer to the ventral midline relative to their position at stage 6. The position of the *Ct-piwi1 *positive ventro-lateral clusters also varies among stage 8 larvae, with the following observed patterns: (1) bilaterally symmetric clusters at the midline (n = 15/35), (2) bilaterally symmetric clusters lateral to the midline (n = 11/35), (3) asymmetrically positioned clusters lateral to the midline, with one of the clusters more anterior to the other (n = 9/35) (Figure 3L, arrows). By stage 9, only a single cluster at the ventral midline of segment 4 is apparent (Figure 3M, arrows). This cluster is positioned dorsal to the ventral nerve cord and ventral to the gut tube, at the boundary between the foregut and midgut (Figure 3N). From stage 6 to stage 9, the position of these *Ct-piwi1*-expressing cells changes from paired ventro-lateral clusters (n = 35/35) to a single cluster at the ventral midline (n = 30/32). The observed variation in the position of the ventro-lateral cell clusters of *Ct-piwi1*-expressing cells and their progression to the ventral midline is consistent with migratory behavior of these cells. There is no obvious increase in the number of cells in these clusters between stage 6 and stage 9. We hypothesize that these cells are PGCs. By stage 9, *Ct-piwi1 *expression is restricted to the posterior growth zone and a single cluster of putative PGCs at the ventral midline.

**Figure 4:**
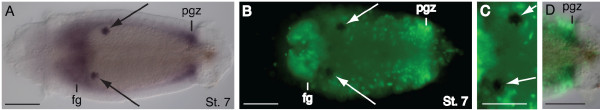
**Pattern of *Ct-piwi1 *expression and EdU incorporation in a stage 7 larva**. Larva were exposed to EdU for one hour. All images are from the same animal and same focal plane. All panels are a ventral view with anterior to the left. Abbreviations: fg, foregut; pgz, posterior growth zone. (**A**) DIC image of a stage 7 larva showing *Ct-piwi1 *expression in the foregut, posterior growth zone, and two asymmetrically positioned ventro-lateral cell clusters (black arrows). (**B**) Fluorescently tagged EdU labeling (green) shows regions of dividing cells in the foregut, and posterior growth zone. Note the absence of dividing cells in the *Ct-piwi1-*expressing asymmetrically positioned ventro-lateral cell clusters (white arrows). (**C**) A close-up view of the *Ct-piwi1 *positive asymmetrically positioned ventro-lateral cell clusters (white arrows) from panel (**B**). (**D**) Combined DIC and fluorescent images in the region of the posterior growth zone showing co-localization of *Ct-piwi1 *expression with EdU incorporation. Scale bar, 50 μm for all panels.

The position of *Ct-piwi1 *expression domains was characterized in relation to patterns of cell division in larvae as visualized by EdU incorporation. A comprehensive study of cell division patterns during larval development of *C. teleta *has been previously reported [27]. *Ct-piwi1 *expression corresponds with regions of dividing cells in *C. teleta *larvae, including in the foregut and posterior growth zone in stage 7 larvae (Figure 4A, B, D). However, there are also EdU positive cells that lack *Ct-piwi1 *expression, and there are *Ct-piwi1 *positive cells that do not appear to be EdU positive (Figure 4A, B, C, arrows). For example, the putative PGCs are not EdU positive, consistent with our observations that the number of cells does not appear to increase during larval stages. Therefore, there is only partial overlap of these two patterns.

**Figure 5:**
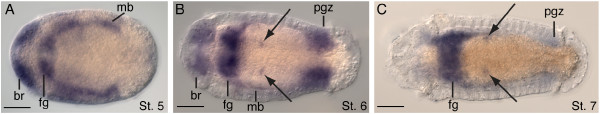
***Ct-piwi2 *expression during larval development**. All animals are a ventral view with anterior to the left. Abbreviations: br, brain; fg, foregut; mb, mesodermal bands; pgz, posterior growth zone. (**A**) Stage 5 larva showing *Ct-piwi2 *expression in the brain, foregut, and mesodermal bands. (**B**) Stage 6 larva shows expression in the two asymmetrical ventro-lateral cell clusters (arrows), brain, foregut, mesodermal bands, and posterior growth zone. (**C**) Expression is in the two asymmetrical ventro-lateral cell clusters (arrows), foregut, and posterior growth zone an early stage 7 larva. Expression in the brain and mesodermal segments has diminished at this stage. Scale bar, 50 μm for all panels.

Expression of *Ct-piwi2 *was also characterized during embryonic and larval development. *Ct-piwi2 *expression patterns overlap with those of *Ct-piwi1 *throughout early cleavage stage embryos (data not shown), and larval stages 5 to 7 (Figure 5). At stage 5, *Ct-piwi2 *is detected in the brain, presumptive foregut and in the mesodermal bands (Figure 5A). At stage 6, brain and mesodermal band expression begins to fade, while foregut and posterior growth zone expression persists (Figure 5B). *Ct-piwi2 *is also expressed in the PGCs at this stage (Figure 5B, arrows). Expression in the segmental trunk continues to diminish during stage 7; however, expression is still evident in the foregut, posterior growth zone, and ventro-lateral cell clusters (Figure 5C, arrows). The expression of *Ct-piwi2 *and and *Ct-piwi1 *in the ventro-lateral cell clusters are also similar to each other at late larval stages when the clusters are localized to the midline (data not shown). *Ct-piwi2 *expression also overlaps with that of *Ct-piwi1 *in adults (see below), and is observed in the PGCs, genital ducts, immature oocytes, and posterior growth zone (data not shown). Although there may be minor differences in expression of *Ct-piwi1 *and *Ct-piwi2*, the expression patterns are largely similar during the stages examined, thus we did not further characterize expression of *Ct-piwi2*.

### *Ct-piwi1 *expression in juveniles

**Figure 6:**
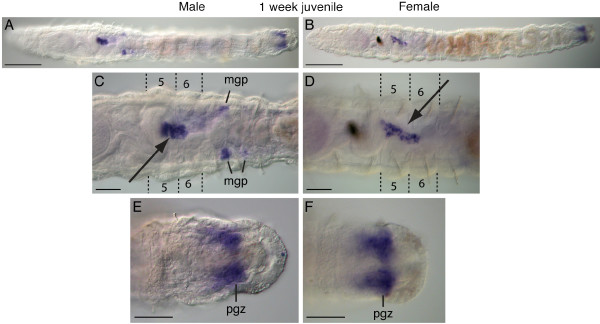
***Ct-piwi1 *expression patterns in one week post-metamorphic juveniles**. All panels are ventral views with anterior to the left. Abbreviations: mgp, male gamete precursor; pgz, posterior growth zone. Dotted lines in C and D indicate the segment boundaries of the 5^th ^and 6^th ^segments. (**A**) Male juvenile worm. (**B**) Female juvenile worm. (**C**) In a male juvenile, *Ct-piwi1 *expression is in the PGCs in the fifth and sixth segments (arrow), and bilaterally symmetrical male gamete precursors at the boundary of the seventh and eighth segments. (**D**) In female juveniles, expression is in the PGCs in the fifth and sixth segments (arrow). (**E, F**) A close-up views of expression in the posterior growth zone in a male (**E**) and female (**F**) juvenile. Scale bar, 50 μm for all panels except **A **and **B**, which are 100 μm.

*Ct-piwi1 *expression was observed in distinct domains in one week post-metamorphic juvenile worms (Figure 6A, B). Within the thoracic region of both males and females (segments 1 to 9), there is expression in a discrete structure of approximately 25 cells that is localized within the coelomic cavity to the ventral midline of segment 5, usually extending into segment 6 (Figure 6C, D, arrows). We think these cells are PGCs. Males and females also express *Ct-piwi1 *in the posterior growth zone (Figure 6E, F). In addition, males have *Ct-piwi1*-expressing cells in a pair of cell clusters positioned at the junction between segments 7 and 8 (Figure 6C). In some animals, a second cluster was also apparent at the junction between and segments 8 and 9. These clusters are in a ventro-lateral position in the coelomic cavity, and based on their location, they are likely to be male gamete precursors. At this stage, the ovaries of the female have not yet developed, and there is only expression in the PGCs and posterior growth zone in females (Figure 6B, D, F).

**Figure 7:**
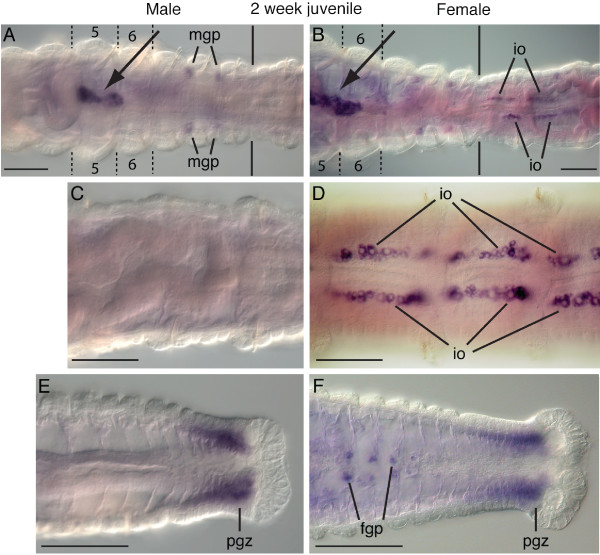
***Ct-piwi1 *expression patterns in two-week post-metamorphic juveniles**. All panels are ventral views with anterior to the left. Abbreviations: mgp, male gamete precursor; io, immature oocytes; pgz, posterior growth zone; fgp, female germline precursors. Dotted lines in **A **and **B **indicate the segment boundaries of the fifth and sixth segments. Black solid lines in **A **and **B **indicate the thoracic abdominal boundary. (**A**) In a male juvenile, *Ct-piwi1 *expression is in the PGCs in the fifth and sixth segments (arrow), and in bilaterally symmetrical male gamete precursors at the boundaries of the seventh and eighth segment and eighth and ninth segment. (**B**) In a female juvenile, expression is in the PGCs (arrow) and immature oocytes in the ovaries, which begin at the 10^th ^segment (the first abdominal segment). (**C**) Male juvenile displaying lack of expression in abdominal segments. (**D**) Female juvenile showing *Ct-piwi1 *expression in immature oocytes in abdominal segments. (**E**) Expression in the posterior growth zone of a male juvenile. (**F**) In a female juvenile, expression is in female germline precursors and the posterior growth zone. Scale bar, 50 μm for all panels.

In two-week post-metamorphic juvenile worms, *Ct-piwi1 *expression domains are similar to those observed in one week post-metamorphic juvenile worms. The appearance of ovaries within the females is the biggest difference between the two stages. The ovaries appear as paired ventral structures adjacent to the lateral edges of the intestine, and at this stage primarily contain previtellogenic oocytes. The most anterior segment that contains ovaries is the 10^th ^segment (Figure 7B), posterior to the thoracic region, and at this stage the ovaries span many segments. *Ct-piwi1 *is expressed in immature oocytes within the ovaries of females (Figure 7B, D), and is not detected within mid-body abdominal segments of males (Figure 7C). In males, *Ct-piwi1*-expressing cells are present in two pairs of ventro-lateral cell clusters positioned at the boundaries between segments 7 and 8 and segments 8 and 9 (Figure 7A). The structure containing the putative PGCs is larger in area and more elongated compared to the structure in one-week post-metamorphic juveniles, and now contains approximately 50 cells, within segments 5 to 6 (Figure 7A, B, arrows). The posterior growth zone of males and females maintains strong *Ct-piwi1 *expression, which is most prominent in the mesoderm (Figure 7E, F). Anterior to the posterior growth zone in females, there are segmentally repeated, paired ventral cell clusters between the ventral nerve cord and gut that express *Ct-piwi1 *(Figure 7F). These clusters are positioned along the anterior face of the septa at the segmental boundary. We hypothesize that these cell clusters are female germline precursors that will colonize the future ovaries once these segments mature and ovaries form within them. In approximately one-third to one-half of the two-week juveniles (n = 13/30), we also observed *Ct-piwi1 *expression in cells scattered in the trunk within the coelomic cavity (not shown). These cells have a large nuclear to cytoplasmic ratio, lack obvious signs of morphological differentiation, and their position is highly variable within the coelomic cavity among individuals. We rarely saw these cells in one-week juveniles and reproductive adults.

### *Ct-piwi1 *adult expression patterns

**Figure 8:**
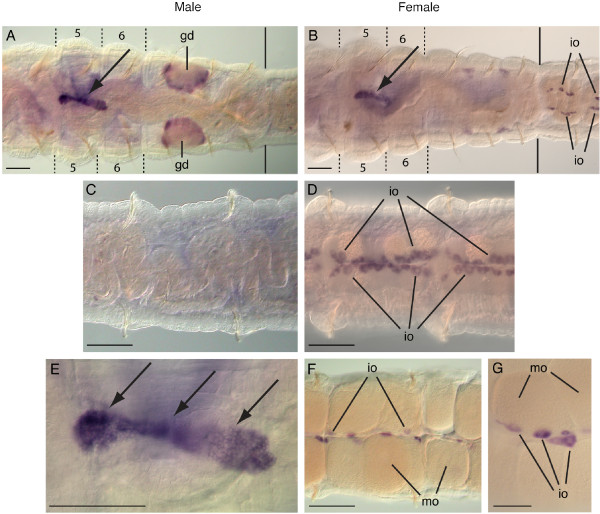
**Adult expression patterns of *Ct-piwi1***. All panels are a ventral view with anterior to the left. Abbreviations: gd, genital duct; io, immature oocytes; mo, mature oocytes. Dotted lines in A and B indicate the segment boundaries of the fifth and sixth segments. Black solid lines in A and B indicate the boundary between the thorax and abdomen. (**A**) *Ct-piwi1 *expression in the PGCs in the fifth and sixth segments of a male (arrow) and bilaterally symmetrical genital ducts spanning the boundary of the seventh and eighth segment. (**B**) Adult hermaphrodite with expression in the PGCs in the fifth and sixth segments (arrow). Oocyte expression is visible in the ovaries in the 10^th ^segment. (**C**) Expression is not detected within abdominal segments of adult males. (**D**) Enlarged view of female showing expression in immature oocytes of abdominal segments. (**E**) Close-up view of *Ct-piwi1*-expressing PGCs (arrows) from panel A. (**F**) Adult female with *Ct-piwi1 *expression in immature oocytes located in the medial region of the ovary. Note the lack of expression in the large mature oocytes. (**G**) Close-up view of expression in immature oocytes within a single abdominal segment. Scale bar, 50 μm for all panels.

Expression of *Ct-piwi1 *was also examined in reproductive adult worms eight weeks post-metamorphosis. The overall expression pattern in adults is similar to that of two-week post-metamorphic juvenile worms. Within the thoracic region (segments 1 to 9) of males and females, *Ct-piwi1 *expression persists in the putative PGCs localized to segment 5. This structure has continued to enlarge and at this stage contains over 75 *Ct-piwi1*-expressing cells, typically extending into segment 6 (Figure 8A, B, E, arrows).

*Ct-piwi1 *is also expressed in the gonads. Adult females express *Ct-piwi1 *in the ovaries in the abdominal segments (Figure 8B, D, F, G). Each ovary contains oocytes at different stages of development [28]. *Ct-piwi1 *is only detected in the medial immature oocytes and not in the large, laterally-positioned mature oocytes within each ovary (Figure 8F, G). Immature oocyte expression is present in many mid-body abdominal segments as clusters of cells adjacent to the ventral midline (Figure 8D). Males express *Ct-piwi1 *in the symmetrical ventro-lateral genital ducts spanning the boundary between segments 7 and 8 (Figure 8A), but there is no detectable expression within the mid-body abdominal segments (Figure 8C). Male and female adults also maintain strong *Ct-piwi1 *expression in the posterior growth zone (data not shown).

### *Ct-piwi1 *expression during regeneration

**Figure 9:**
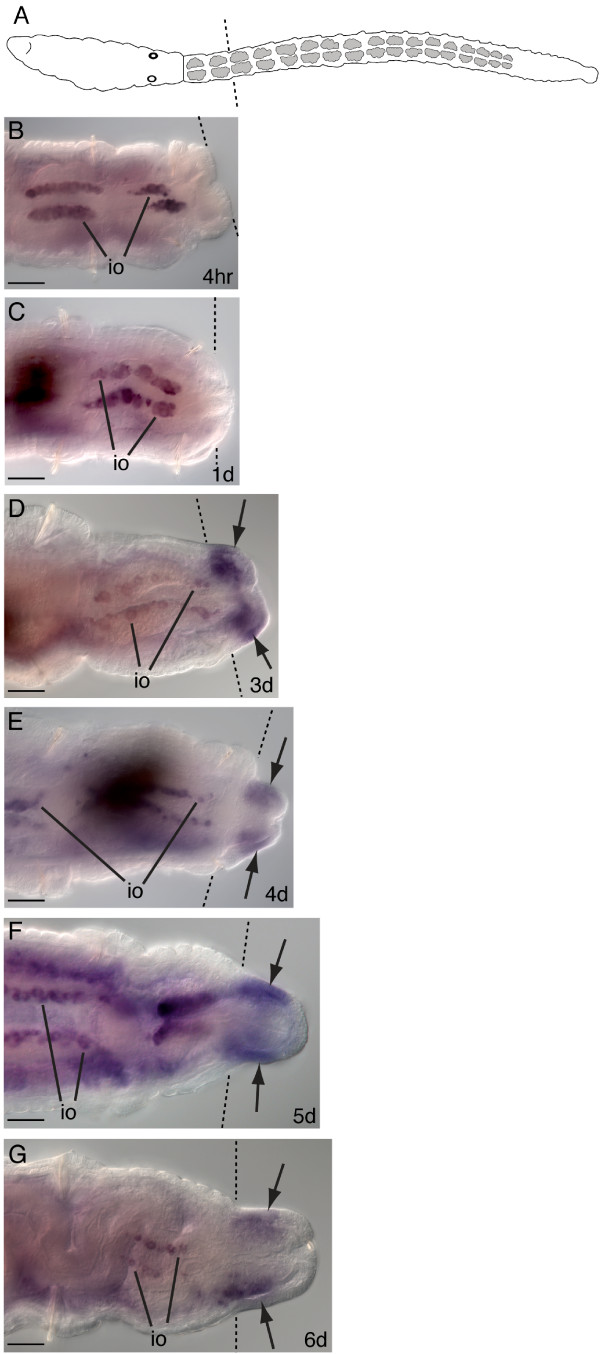
***Ct-piwi1 *expression during early stages of adult regeneration**. All animals are female and were amputated at the 12^th ^segment. All panels shown are posterior ends of amputated adults. All panels are oriented in a ventral view with anterior to the left. Dotted lines indicate the cut site in all panels. Expression in immature oocytes of the pre-existing tissue is abbreviated: io. Dark shapes in **C **and **E **are contents within the lumen of the gut. (**A**) Schematic of an adult worm indicating the cut site at the 12^th ^segment. (**B**) Posterior end of an adult four hours post-amputation. (**C**) One day post-amputation showing complete wound healing. (**D**) Three days post-amputation shows *Ct-piwi1 *expression in the regenerating blastema (arrows). (**E**) Four days post-amputation showing expression in the blastema (arrows). (**F**) Expression is in the blastema five days post-amputation (arrows). (**G**) Six days post-amputation with expression in the anterior and mid-region of the blastema (arrows). Scale bar, 50 μm for all panels.

Amputations were made on adult female worms at the segment boundary between the 11^th ^and 12^th ^segments, and *Ct-piwi1 *expression was monitored at different time points during the course of regeneration. A schematic shows the location of the cut site at the 12^th ^segment (Figure 9A, dotted line). At all stages examined, expression of *Ct-piwi1 *is maintained in the pre-existing tissue in the gonads and putative PGCs during regeneration. Following amputation, wound healing occurs within four hours post-amputation (Figure 9B). At one day post-amputation, the wound has fully healed; *Ct-piwi1 *is not expressed in the blastema at this point or during wound healing (Figure 9B, C). The earliest *Ct-piwi1 *expression is detectable in the regenerating tissue at three days post-amputation, during growth of the blastema and prior to the appearance of segments (Figure 9D, arrows). Expression is present in both the mesoderm and ectoderm, and is more pronounced on the ventral side of the blastema. As the blastema continues to grow (four to six days post-amputation), *Ct-piwi1 *expression persists in the regenerating tissue and is most prominent in the ventral mesoderm (Figure 9E, F, G, arrows). At these stages, it becomes clear that *Ct-piwi1 *is present in the proximal and mid-portion of the regenerate, but is absent from the most posterior end.

**Figure 10:**
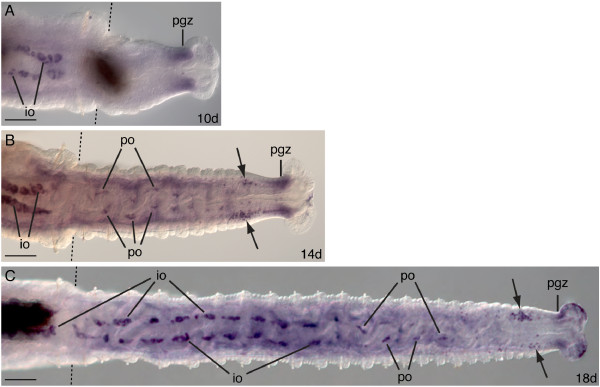
**Expression of *Ct-piwi1 *in late stage adult regenerates**. All animals are female and were amputated at the 12^th ^segment. All panels shown are posterior ends of amputated adults. All panels are oriented in a ventral view with anterior to the left. Abbreviations: io, immature oocytes; pgz, posterior growth zone; po, putative oocytes. Dotted lines indicate the cut site in all panels. Dark shapes in A and C are contents within the lumen of the gut. (**A**) At 10 days post-amputation, expression is in the posterior growth zone of the regenerating tissue. (**B**) 14 days post-amputation showing expression in putative oocytes, the posterior growth zone and loosely organized cells within the coelomic cavity (arrows). (**C**) Expression in 18 days post-amputation is in immature oocytes in regenerating ovaries, putative oocytes, cells within the coelomic cavity (arrows), and the posterior growth zone. Scale bar, 50 μm for all panels.

At later stages of regeneration, *Ct-piwi1 *expression becomes more restricted. In 10 through 18 days post-amputation, there is a morphologically distinct pygidium and posterior growth zone. During these stages, *Ct-piwi1 *is consistently expressed in the posterior growth zone of the regenerating tissue (Figure 10A-C), and at 10 days post-amputation, it is the most prominent expression domain. At 14 days post-amputation, segments become apparent externally and additional expression domains appear, including in a loosely organized group of cells anterior to the posterior growth zone in the ventro-lateral region of the coelomic cavity (Figure 10B, arrows). In addition, in the anterior segments of the regenerating animals, there is expression associated with the ventral face of the gut in the mesoderm; this domain corresponds to the position where the ovaries will form. We interpret these *piwi*-expressing cells to be oogonia. Approximately 20 segments have regenerated by 18 days post-amputation. At this stage, ovaries have begun to form in the anterior segments of the regenerate, and they contain *piwi*-expressing immature oocytes (Figure 10C). In the middle segments of the regenerate, *Ct-piwi1 *is expressed in a pattern very similar to that observed in anterior regenerating segments of 10-day post-amputation adults, in putative oogonia. Expression is also evident in loosely organized cells anterior to the posterior growth zone in the coelomic cavity (Figure 10C, arrows). Similar amputations were also performed on adult males, and there were no detectable differences in *Ct-piwi1 *expression between males and females within the regenerating tissue from zero to ten days post-amputation (not shown). Moreover, between 14 and 18 days post-amputation, both males and females exhibit similar expression in the posterior growth zone and in a population of loosely organized cells anterior of the posterior growth zone. In summary, there are two distinct phases of *Ct-piwi1 *expression during regeneration: an early phase in a broad domain during blastemal growth, and a later phase of more restricted expression in the posterior growth zone, regenerating ovaries (of females and hermaphrodites), and in a localized population of loosely organized cells in the coelomic cavity.

## Discussion

### Expression of *piwi *in germline and somatic tissues of *C. teleta*

*Ct-piwi1 *and *Ct-piwi2 *are expressed throughout the life history of *C. teleta *in a dynamic spatial pattern that includes both somatic and germline cells. The two genes show very similar expression patterns to each other. Both genes are broadly expressed during embryonic and early larval development, and gradually become restricted to the posterior growth zone, putative PGCs, and gonads during juvenile and adult stages. In larval stages, the position of the putative PGCs that express *Ct-piwi1 *and *Ct-piwi2 *closely corresponds with the position of cell cluster descendants of the blastomere 4d [9]. Thus, it is likely that the PGCs arise from 4d in *C. teleta*. By examining *Ct-piwi1 *expression at several juvenile stages, we were able to characterize the gradual expansion of the putative PGCs from a small cluster of approximately 4 to 10 cells in late stage larvae to a cluster of over 75 cells in the reproductive adult. The localization of a putative PGC population to a structure located in segment 5 of juveniles may serve as a transient intermediate target for PGCs or a PGC niche prior to adult gonad formation. In many animals, PGCs form far from the gonads and migrate to the sites of developing ovaries or testes in the embryo [29]. Migration of PGCs has also been reported in a few polychaetes [11,23]. We hypothesize that in older juveniles of *C. teleta*, some of the PGCs migrate from this niche to the gonads in segments 7 and 8 of males and to the reproductive abdominal segments in females. Our observation of scattered *Ct-piwi1 *expressing cells in the coelomic cavity of juveniles at a stage when the gonads begin to form (two weeks post-metamorphosis) is consistent with this hypothesis.

### Localization of putative PGCs in *C. teleta *adult worms

A surprising result from our characterization of *Ct-piwi *gene expression patterns, and also from previous analyses of *vasa *and *nanos *gene orthologs in *C. teleta *[24] is the identification of a population of putative PGCs that persist in sexually mature adults, even after the gonads have formed and contain mature gametes. These putative PGCs are encased by a thin sheath of cells and suspended by mesenteries on the ventral side of the coelomic cavity, spanning segment 5 and partially into segment 6. This structure was not previously identified by TEM/morphological studies. In contrast to many segmentally-repeated features in the mid-body, this cluster of putative PGCs is present as a single structure. A similar structure called the 'primary gonad' has been described in *P. dumerilii *[23]; however, because we did not observe any steps of gametogenesis in this structure, we do not adopt the term 'gonad'. Instead, our observations are more consistent with referring to this structure as a PGC niche. This niche appears to be a permanent structure that provides a source of new PGCs throughout the animal's life, and it is located separate from the gonads. Although the biological significance of maintaining such a population of PGCs in mature adults is currently unknown, there are several features of the reproductive biology of *C. teleta *that may require a persistent stock of PGCs. Both males and females can reproduce multiple times and these PGCs may re-populate the gonads to ensure the propagation of multiple generations. Additionally, since ovaries can reform following amputation of abdominal segments, cells from this PGC population may colonize the regenerating ovaries. Furthermore, since sexually mature males can be environmentally induced to produce oocytes as hermaphrodites [6], maintaining a reservoir of PGCs may permit such phenotypic plasticity of the sexes and provide a source of germ cells to populate newly forming ovaries. Thus, this system provides a good opportunity to study the epigenetic control of gamete determination and phenotypic differentiation.

### Origin of the germline during regeneration in *C. teleta*

The origin of the oocytes that populate regenerating ovaries following amputation in *C. teleta *is currently unknown. As previously hypothesized, oocyte precursors may originate from a resident PGC population in segment 5 (pre-existing tissue), and migrate in a posterior direction through numerous segments to nascent ovaries within the regenerating tissue [24]. This strategy would provide a rapid supply of germ cell precursors to the body of the regenerating worm. However, our examination of *Ct-piwi1 *expression during regeneration has led us to consider an alternate explanation, and is based on the observation that *Ct-piwi*-positive cells appear *de novo *in the coelomic cavity of regenerating segments when the ovaries begin to form (14 to 18 days post-amputation) (Figure 10B, C). In this scenario, oocytes may originate from multipotent stem cells within the regenerating tissue that later acquire a germ cell identity. We suggest that these germ cells arise from the lining of the coelomic cavities. Such a scenario is consistent with an epigenetic mode of PGC specification in which, following amputation, a signalling event induces somatic stem cells to produce germ cells that then differentiate into oocytes. If this were true, it would indicate that *C. teleta *has the ability to replace lost germ cells, and contrasts with the situation in the model organisms *D. melanogaster*, *Caenorhabitis elegans*, *Danio rerio*, and mice in which ablation of the germline results in sterile animals [13,14,16].

Since many model systems have a fully segregated germline and lack regenerative capabilities, detailed studies of these animals may have disproportionately influenced our views concerning the segregation of the germline from the soma. Multipotent stem cells in some bilaterian animals retain the ability to generate germline cells [3]. Under normal circumstances in such animals, PGCs are segregated from somatic tissues during early development and are responsible for generating all gametes. In altered conditions, such as during regeneration or when PGCs are experimentally removed, multipotent stem cells in somatic tissue may compensate and produce gametes. For example, during normal development in the ascidian *Ciona intestinalis*, germ cells in the tailbud of the tadpole stage are absorbed during metamorphosis and persist as PGCs in the young juvenile. However, upon removal of the larval tail prior to metamorphosis, PGCs from another source appear in the gonad rudiment at a later stage [30]. Following removal of *vasa*-expressing micromeres in the embryo of the sea urchin *Strongylocentrotus purpuratus*, an accumulation of Vasa protein is induced in other cells that presumably give rise to functional PGCs [31]. These observations indicate the presence of a compensatory mechanism to produce PGCs from somatic stem cells in the absence of the original germ cells.

Although *piwi *is best known for its role in the germline, there are a growing number of cases in which expression has also been reported outside the gonads and the germline, often in multipotent stem cells. Examples include not only early branching metazoans such as sponges [32,33], hydrozoan cnidarians [21] and ctenophores [20], but also bilaterians such as acoel flatworms [34], planarians [35,36], tunicates [30], and another polychaete annelid [23]. Our observations of *Ct-piwi1 *and *Ct-piwi2 *expression in both the germline and in regions of dividing cells in the posterior growth zone add another example to this list. Recently, *piwi *and *vasa *genes have been proposed to be ancestrally associated with stem cell character ('stemness'), rather than solely with germline stem cells [20]. In animal lineages such as in the mussel *Mytilus galloprovincialis*, in which there is restricted expression *vasa *in the germline [37], there could have been a partial or complete loss of expression of this gene from somatic stem cell lineages. In *C. teleta*, *vasa*, *nanos *and *piwi *orthologs are all expressed in very similar patterns to one another in both the germline and posterior growth zone, likely in multipotent stem cells.

### Comparison of *piwi *expression patterns and PGC migration among annelids

The patterns of *Ct-piwi1 *and *Ct-piwi2 *expression in *C. teleta *show both similarities and differences when compared to *piwi *expression in the two other annelids that have been examined. In both *C. teleta *and the polychaete *P. dumerilii*, *piwi *is detected in PGCs and the posterior growth zone in larval and juvenile stages [23]. In *C. teleta *larvae, the PGCs intitially appear several segments anterior to the posterior growth zone, in the mid-trunk segments. In *P. dumerilii*, although the PGCs arise from the mesoderm in the posterior growth zone, at the end of larval development and in juvenile stages, the PGCs migrate anteriorly, and following generation of additional segments, to the primary gonad. It has been proposed that the primary gonad serves as an intermediate residence for PGCs in *P. dumerilii *[23]. Interestingly, juveniles of both polychaetes express *piwi *in a population of PGCs in segment 5, the location of the primary gonad in *P. dumerilii*. However, there is an important distinction between these two structures at this location. The PGC population in *C. teleta *is positioned on the ventral side of the coelomic cavity, whereas in *P. dumerilii*, it is on the dorsal side of the body in a circumferential band. In contrast to *C. teleta*, there are no somatic gonads in *P. dumerili*. Instead, oocytes mature within the coelomic cavity, initially as clusters and later singly as individual oocytes [28,38,39]. As *P. dumerili *juveniles mature, PGCs migrate from the primary gonad to the base of the segmentally iterated parapodia in both males and females, where gonial clusters later appear.

*Piwi *expression has also been described in the oligochaete *E. japonesis*. This species normally reproduces asexually and can be induced to undergo sexual reproduction under starvation conditions. In the asexual phase, *Ej-piwi *is expressed in cells distributed throughout the body. During starvation conditions, gonads form in the seventh and eighth segments and *Ej-piwi *is expressed in the developing gonads [22], similar to gonad expression in reproductive adults of *C. teleta*. During regeneration, *piwi *expression patterns are distinct between *E. japonesis *and *C. teleta*. In *C. teleta, piwi *is expressed during blastemal growth, and later, in a more restricted pattern following differentiation in the regenerating segments. This later phase includes *piwi*-expressing cells in the nascent ovaries of the regenerated tissue. In contrast, expression is not detected within the blastema during early stages of regeneration in *E. japonensis*. Instead, following amputation, discrete *piwi*-expressing cells localize to the region of the amputation site, proximal to the blastema. Later, as regenerating tissues begin to differentiate and segments form in *E. japonesis*, *piwi*-expressing cells appear in the regenerate and eventually become localized to the sites of the forming gonads. In summary, although there are clear species-specific differences in reproductive anatomy and morphogenesis, it appears that in annelids there is conservation of *piwi *expression in the primordial germ cells, developing gametes, and posterior growth zone.

## Conclusions

The expression of *Ct-piwi1 *and *Ct-piwi2 *in both the germline and regions of dividing cells in the posterior growth zone provides a molecular link between germline stem cells and pluripotent somatic stem cells in *C. teleta*. Furthermore, the similarity in expression of *Ct-piwi1 *to the expression patterns previously observed for *vasa *and *nanos *homologs in *C. teleta *[24], suggests that this core set of stem cell regulators has retained an ancestral role in somatic and germline stem cell production. Such a dual role may reflect an ancestral metazoan feature in which there was a close link between somatic and germline stem cells, and contrasts with the segregation of the germline in animals such as *D. melanogaster *[40], *C. elegans *[41] and *D. rerio *[42].

## Materials and methods

### Cloning of *Capitella teleta piwi1 *and *piwi2 *genes

Several overlapping expressed sequence tag (EST) sequences representing a single *piwi1 *homolog (*Ct-piwi1*) and another set representing a single *piwi2 *homolog (*Ct-piwi2*) were identified in BLAST searches of *Capitella *EST libraries from the *C. teleta *8x genome sequencing project (Joint Genome Institute, Department of Energy, Walnut Creek, CA, USA, http://genome.jgi-psf.org/Capca1/Capca1.home.html). Each set of sequences was aligned and compiled into a single predicted transcript for each gene. EST clones containing both *Ct-piwi1 *and *Ct-piwi2 *fragments from a mixed stage plasmid cDNA library were streaked on LB-ampicillin plates from -80°C glycerol stocks. Both *Ct-piwi *clones were checked for correct insert sizes, and sequenced for verification (Macrogen, Seoul, South Korea). The predicted transcripts were submitted to the National Center for Biotechnology Information (NCBI) as third-party annotation sequences with the following accession numbers: *Ct-piwi1 *(BK007975) and *Ct-piwi2 *(BK007976). The *Ct-piwi1 *riboprobe is approximately 1.5 kb and spans nearly the entire conserved PIWI domain, which consists of about 900 bp. The *Ct-piwi2 *riboprobe is 920 bp and includes the 3' end of the PIWI domain as well as 3' untranslated region. The two probes overlap by about 300 bp and have a sequence similarity in this region of 64%.

### Sequence alignments and phylogenetic analysis

tBLASTn searches of the *C. teleta *genome were conducted to find all homologs of *D. melanogaster *Piwi (NCBI accession number NP_476875.1). Two putative orthologs were found in the *C. teleta *genome corresponding to JGI protein IDs 154759 and 163584. Two additional sequence hits were examined and did not include the PAZ domain characteristic of Piwi proteins and were not included in further analyses.

Amino acid sequences for related proteins across a broad diversity of animal taxa were downloaded from the protein database in GenBank. Additional lophotrochozoan sequences were obtained from the genomes of *Lottia gigantea *and *Helobdella robusta *(Joint Genome Institute, Department of Energy, Walnut Creek, CA, USA, http://genome.jgi-psf.org/). The conserved PAZ (Piwi Argonaut Zwille) and PIWI domains were identified by a Pfam search using default parameters. Only the PIWI domain was used to create an amino acid sequence alignment due to the high divergence rates of the PAZ domain. The 327 amino acid alignment was created with ClustalX using default parameters in MacVector v11.0 and hand corrected for obvious alignment errors.

ProtTest v2.4 [43] was used to determine the appropriate model of protein evolution. The RtRev model was recommended and used for both Bayesian and maximum likelihood analyses. Bayesian analysis was conducted with MrBayes v3.1.2 [44] A total of 3,000,000 generations were run, sampled every 100 generations, with four independent runs and four chains. Once convergence was reached, a majority rule consensus tree was generated with burnin of 8,100 trees. Maximum likelihood analysis was performed with PhyML 3.0 [45] using the RtRev model with 1,000 bootstrap replicates.

Trees were visualized in FigTree 1.3.1 [46] and drawn using Adobe Illustrator version CS4. GenBank and Swiss-Prot accession numbers and protein identification numbers from JGI are listed (Additional file 1). The nexus alignment is available upon request.

### Animal husbandry

A *C. teleta *colony was maintained in the laboratory at 19°C according to published culture methods [47]. Juvenile and adult worms were maintained in bowls of 20-μm filtered sea water (FSW) and provided with sieved ocean mud as a food source. Parental brood tubes were recovered by sifting mud through a fine-mesh sieve. Embryos and larvae were dissected from brood tubes and raised to the desired stage.

### Amputations

Regeneration experiments were performed on mature adults at eight weeks post-metamorphosis. Adults are sexually mature at 8 to 10 weeks when raised at 19°C. Animals were removed from the mud and placed in a dish of FSW for two hours to allow them to excrete ingested material. Animals were then relaxed for 20 minutes in 0.37 M MgCl_2_. Individual worms were placed on a strip of dental wax (Electron Microscopy Sciences, Hatfield, PA, USA) in one to three drops of FSW. With the aid of a dissecting microscope (Zeiss, Gottingen, Germany), all amputations were made at the anterior edge of the 12^th ^segment using a microsurgery scalpel (#715, Feather Safety Razor Co., Osaka, Japan). Posterior tissue segments were discarded. Following amputation, animals were returned to a separate 35 mm or 60 mm dish with FSW and left overnight. After 24 hours, mud was added to the dish and animals were maintained at 19°C. Specimens were periodically removed from the mud to monitor regeneration. Animals were fixed at different time points following amputation in 3.7% formaldehyde in FSW at 4°C for 16 to 24 h and then processed either for morphological analysis, immunohistochemistry or whole-mount *in situ *hybridization (see below).

### Whole-mount *in situ *hybridization

Embryos (stages 1 to 3) were pretreated in a 1:1 mixture of 1.0 M sucrose and 0.25 M sodium citrate (Sigma-Aldrich Co., St. Louis, MO, USA) for three minutes, washed in FSW, and fixed in 3.7% formaldehyde in FSW overnight at 4°C. Larvae (stages 4 to 9), juveniles (one and two weeks post-metamorphosis), adults and amputated adults (eight weeks post-metamorphosis) were relaxed in 1:1 0.37 M MgCl_2_:FSW for 10 minutes and fixed in 3.7% formaldehyde/FSW overnight at 4°C. Embryos, larvae, juveniles and adults were then washed in phosphate-buffered saline (PBS), dehydrated in methanol and stored at -20°C. Whole-mount *in situ *hybridization followed published protocols [48,49]. Juveniles and adults were treated with the same conditions as embryos and larvae with the exception that proteinase K treatment was increased from 5 minutes to 10 minutes for juveniles and to 20 minutes for adults and amputated adults. In addition, for juvenile, adult and amputated adult stage experiments, the volume of all washes and hybridizations was increased from 0.5 to 1 ml. Digoxigenin-labeled riboprobes for *Ct-piwi1 *and *Ct-piwi2 *were generated with the MEGAscript kit (Ambion, Inc., Austin, TX, USA). For embryos, larvae, and one-week post-metamorphic juveniles, the *Ct-piwi1 *and *Ct-piwi2 *probe concentration was 1.0 ng/μl. For two-week post-metamorphic juveniles and adults, *Ct-piwi1 *and *Ct-piwi2 *were used at a concentration of 0.5 ng/μl. Following hybridization, probes were detected using nitroblue tetrazolium chloride/5-bromo-4chloro-3indolyphosphate (NBT/BCIP) color substrate. Typically, the color reaction was allowed to develop between one hour and three days; however, in uncleaved zygotes, two-cell and four-cell stage embryos a reaction between 6 and 11 days was necessary to detect transcripts. Specimens were equilibrated in glycerol (80% glycerol/10% 10x PBS/10% diH_2_O) and mounted on Rainex^®^-coated slides.

### Microscopy

Microscopic analyses were performed on a Zeiss Axioskop 2 compound light microscope (Zeiss, Gottingen, Germany). Micrographs were captured with a stem-mounted SpotFlex digital camera (Diagnostic Instruments, Inc., Sterling Heights, MI, USA). Multiple DIC focal planes were merged for some images using Helicon Focus (Helicon Soft Ltd., Kharkov, Ukraine). Confocal imaging was done using a Zeiss LSM 710 confocal microscope and Z-stack projections were generated using the ImageJ software (NIH).

### EdU labeling

To detect dividing cells, the Click-iT EdU imaging kit was used to label cells undergoing DNA synthesis (Invitrogen Co., Carlsbad, CA, USA). The kit protocol was followed except for the following modifications. Stage 5 to 7 larvae were incubated for one hour in 300 μM 5-ethynyl-2'-deoxyuridine (EdU) in FSW (10 mM working stock diluted in PBS), and then fixed overnight at 4°C, dehydrated in methanol. *In situ *hybridization experiments were performed according to the methods described above. Following the NBT/BCIP probe detection step, animals were washed in the following: 2X in PBS, 1X in PBS + 0.5% Triton for 20 minutes, and 2X in PBS + 3% BSA for 5 minutes each. Subsequently, the EdU detection reaction was carried out, but reduced to a total volume of 200 μl. Animals were rinsed several times with PBS, equilibrated in 80% glycerol, and analyzed and imaged following the methods for *in situ *hybridization described above.

### Immunohistochemistry

Following fixation, regenerating adults were rinsed in PBS + 0.5% Triton, and permealibilized by incubation with 0.01 mg/ml proteinase K (Invitrogen) for 20 minutes. Specimens were then re-fixed for 20 minutes in 4% paraformaldehyde in PBS. Following 3X washes in PBS over 10 minutes and 2X washes in PBS + 0.5% Triton, animals were blocked for 2 hours at room temperature in PBS + 0.5% Triton + 10% normal goat serum (Sigma). Animals were incubated overnight at 4°C in a 1:400 dilution of anti-acetylated tubulin antibody (Sigma) followed by several washes in PBS + 0.5% Triton, and incubation in a 1:400 dilution of anti-mouse Alexa Fluor 488 secondary antibody (Invitrogen) overnight at 4°C. After washing out the secondary antibody for three hours at room temperature in PBS + 0.5% Triton, animals were cleared overnight in 80% glycerol and analyzed and imaged as described above.

## Abbreviations

BCIP: 5-Bromo-4-cloro-3-indolyl phosphate; BLAST: basic local alignment search tool; BSA: bovine serum albumin; DIC: differential interference contrast; EST: expressed sequence tag; FSW: filtered sea water; JGI: Joint Genome Institute; NBT: nitro blue tetrazolium chloride; NCBI: National Center for Biotechnology Information; PAZ: Piwi Argonaut Zwille; PBS: phosphate buffered saline

## Competing interests

The authors declare that they have no competing interests.

## Authors' contributions

VCG performed *in situ *hybridization experiments, EdU experiments, microscopic analyses and imaging, figure preparation and contributed to writing of the manuscript. ECS carried out immunohistochemistry, contributed to imaging, critical analyses of the data, writing of the manuscript, and designed the study. EY carried out gene orthology analyses, contributed to generation of the *Ct-piwi2 *riboprobe and writing of the manuscript. MJB contributed to synthesis of the *Ct-piwi1 *riboprobe, amputation methods, *in situ *hybridization, gene expression analyses and imaging. All authors contributed to editing of the manuscript and all authors read and approved this manuscript.

## Supplementary Material

Additional file 1 **Accessions**. This file includes a table of the GenBank and Swiss-Prot accession numbers used for sequence alignments and phylogenetic analysis. Also included are Joint Genome Institute protein identification numbers for sequences from the genomes of *C. teleta*, *L. gigantea*, and *H. robusta*. Click here for file

## References

[B1] Yuan H Yamashita YM Germline stem cells: stems of the next generation Curr Opin Cell Biol 2010 22 730 736 10.1016/j.ceb.2010.08.013 20817500 PMC2993778

[B2] Extavour CG Akam M Mechanisms of germ cell specification across the metazoans: epigenesis and preformation Development 2003 130 5869 5884 10.1242/dev.00804 14597570

[B3] Extavour C Evolution of the bilaterian germ line: lineage origin and modulation of specification mechanisms Integr Comp Biol 2007 47 770 785 10.1093/icb/icm027 21669758

[B4] Bely AE Distribution of segment regeneration ability in the Annelida Integr Comp Biol 2006 46 508 518 10.1093/icb/icj051 21672762

[B5] Blake JA Grassle JP Eckelbarger KJ *Capitella teleta *a new species designation for the opportunistic and experimental *Capitella *sp. I with a review of the literature for confirmed records Zoosymposia 2009 2 25 53

[B6] Holbrook MJL Grassle JP The effect of low density on the development of simultaneous hermaphroditism in male *Capitella *species I (Polychaeta) Biol Bull 1984 166 103 109 10.2307/1541434

[B7] Eckelbarger KJ Grassle JP Ultrastructural differences in the eggs and ovarian follicle cells of the *Capitella *(Polychaeta) sibling species Biol Bull 1983 165 379 393 10.2307/1541203 28368231

[B8] Eckelbarger KJ Grassle JP Spermatogenesis, sperm storage and comparative sperm morphology in nine species of *Capitella*, *Capitomastus *and *Capitellides *(Polychaeta: Capitellidae) Mar Biol 1987 95 415 429 10.1007/BF00409572

[B9] Meyer NP Boyle MJ Martindale MQ Seaver EC A comprehensive fate map by intracellular injection of identified blastomeres in the marine polychaete *Capitella teleta* Evo Devo 2010 1 8 10.1186/2041-9139-1-8 PMC2949861 20849573

[B10] Schroeder PC Hermans CO Giese AC, Pearce JS Annelidia: Polychaeta. Reproduction of Marine Invertebrates 1975 III New York: Academic Press 1 213

[B11] Postwald HE Abdominal segment formation in *Spirorbis moerchi *(Polychaeta) Zoomorphology 1981 97 225 245 10.1007/BF00310278

[B12] Saito K Nishida KM Mori T Kawamura Y Miyoshi K Nagami T Siomi H Siomi MC Specific association of Piwi with rasiRNAs derived from retrotransposon and heterochromatic regions in the *Drosophila *genome Genes Dev 2006 20 2214 2222 10.1101/gad.1454806 16882972 PMC1553205

[B13] Seto AG Kingston RE Lau NC The coming of age for Piwi proteins Mol Cell 2007 26 603 609 10.1016/j.molcel.2007.05.021 17560367

[B14] Thomson T Lin H The biogenesis and function of Piwi proteins and piRNAs: progress and prospect Annu Rev Cell Dev Biol 2009 25 355 376 10.1146/annurev.cellbio.24.110707.175327 19575643 PMC2780330

[B15] Ewen-Campen B Schwager EE Extavour CGM The molecular machinery of germ line specification Mol Reprod Dev 2010 77 3 18 19790240 10.1002/mrd.21091

[B16] Cox DN Chao A Lin H *Piwi *encodes a nucleoplasmic factor whose activity modulates the number and division rate of germline stem cells Development 2000 127 503 514 10631171 10.1242/dev.127.3.503

[B17] Lin H Spradling AC A novel group of pumilio mutations affects the asymmetric division of germline stem cells in the *Drosophila *ovary Development 1997 124 2463 2476 9199372 10.1242/dev.124.12.2463

[B18] Tan CH Lee TC Weeraratne SD Korzh V Lim TM Gong Z Ziwi, the zebrafish homologue of the Drosophila piwi: co-localization with vasa at the embryonic genital ridge and gonad-specific expression in the adults Mech Dev 2002 119 S221 S224 14516689 10.1016/s0925-4773(03)00120-5

[B19] Juliano CE Voronina E Stack C Aldrich M Cameron AR Wessel GM Germ line determinants are not localized early in sea urchin development, but do accumulate in the small micromere lineage Dev Biol 2006 300 406 415 10.1016/j.ydbio.2006.07.035 16970939

[B20] Alié A Leclère L Jager H Dayraud C Chang P Le Guyader H Quéinnec E Manuel M Somatic stem cells express *Piwi *and *Vasa *genes in an adult ctenophore: ancient association of "germline genes" with stemness Dev Biol 2011 350 183 197 10.1016/j.ydbio.2010.10.019 21036163

[B21] Seipel K Yanze N Schmid V The germ line and somatic stem cell gene *Cniwi *in the jellyfish *Podocoryne carnea* Int J Dev Biol 2004 48 1 7 10.1387/ijdb.15005568 15005568

[B22] Tadokoro R Sugio M Kutsuna J Tochinai S Takahashi Y Early segregation of germ and somatic lineages during gonadal regeneration in the annelid *Enchytraeus japonensis* Curr Biol 2002 16 1012 1017 10.1016/j.cub.2006.04.036 16713959

[B23] Rebscher N Zelada-González F Banisch TU Raible F Arendt D *Vasa *unveils a common origin of germ cells and of somatic stem cells from the posterior growth zone in the polychaete *Platynereis dumerilii* Dev Biol 2007 306 599 611 10.1016/j.ydbio.2007.03.521 17467683

[B24] Dill KK Seaver EC *Vasa *and *nanos *are coexpressed in somatic and germ line tissue from early embryonic cleavage stages through adulthood in the polychaete Capitella sp. I Dev Genes Evol 2008 218 453 463 10.1007/s00427-008-0236-x 18651171

[B25] Cerutti L Mian N Bateman A Domains in gene silencing and cell differentiation proteins: the novel PAZ domain and redefinition of the Piwi domain Trends Biochem Sci 2000 25 481 482 10.1016/S0968-0004(00)01641-8 11050429

[B26] Hill DH Savage RM Shain DH Evolution development and ecology of *Capitella *sp. I: a waxing model for polychaete studies Annelids in Modern Biology 2009 Hoboken, New Jersey: John Wiley & Sons, Inc 88 115

[B27] Seaver EC Thamm K Hill SD Growth patterns during segmentation in the two polychaete annelids, *Capitella *sp. I and *Hydroides elegans*: comparisons at distinct life history stages Evol Dev 2005 7 312 326 10.1111/j.1525-142X.2005.05037.x 15982368

[B28] Eckelbarger KJ Linley PA Grassle JP Role of ovarian follicle cells in vitellogenesis and oocyte resorption in *Capitella *sp. I (Polychaeta) Mar Biol 1984 9 33 144

[B29] Molyneaux K Wylie C Primordial germ cell migration Int J Dev Biol 2004 48 537 544 10.1387/ijdb.041833km 15349828

[B30] Takamura K Fujimara M Yamagushi Y Primordial germ cells originate from the endodermal strand cells in the ascidian *Ciona intestinalis* Dev Genes Evol 2002 212 11 18 10.1007/s00427-001-0204-1 11875652

[B31] Voronina E Lopez M Juliano CE Gustafson E Song JL Extavour C George S Oliveri P McClay D Wessel G Vasa protein expression is restricted to the small micromeres of the sea urchin but is inducible in other lineages early in development Dev Biol 2008 31 276 286 10.1016/j.ydbio.2007.11.039 PMC2692673 18191830

[B32] Funayama N The stem cell system in demosponges: insights into the origin of somatic stem cells Dev Growth Diff 2010 52 1 14 10.1111/j.1440-169X.2009.01162.x 20078651

[B33] Funayama N Nakatsukasa M Mohri K Masuda Y Agata K Piwi expression in archeocytes and choanocytes in demosponges: insights into the stem cell system in demosponges Evol Dev 2010 12 275 287 10.1111/j.1525-142X.2010.00413.x 20565538

[B34] De Mulder K Kuales G Pfister D Willems M Egger B Salvenmoser W Thaler M Gorny AK Hrouda M Borgonie G Ladurner P Characterization of the stem cell system of the acoel *Isodiametra pulchra* BMC Dev Biol 2009 9 69 10.1186/1471-213X-9-69 20017953 PMC2806412

[B35] Reddien PW Oviedo NJ Jennings JR Jenkin JC Sánchez-Alvarado A SMEDWI-2 is a PIWI-like protein that regulates planarian stem cells Science 2005 310 1327 1330 10.1126/science.1116110 16311336

[B36] De Mulder K Pfister D Kuales G Egger B Salvenmoser W Willems M Steger J Fauster K Micura R Borgonie G Ladurner P Stem cells are differentially regulated during development, regeneration and homeostasis in flatworms Dev Biol 2009 334 198 212 10.1016/j.ydbio.2009.07.019 19631639

[B37] Obata M Sano N Kimata S Nagasawa K Yoshizaki G Komaru A The proliferation and migration of immature germ cells in the mussel, *Mytilus galloprovincialis*: observation of the expression pattern in the *M. galloprovincialis *vasa-like gene (*Myvlg*) by *in situ *hybridization Dev Genes Evol 2010 220 139 149 10.1007/s00427-010-0335-3 20725841

[B38] Fischer A The structure of symplasmic early oocytes and their enveloping sheath cells in the polychaete, *Platynereis dumerilii* Cell Tissue Res 1975 160 327 343 1149120 10.1007/BF00222043

[B39] Fischer A Dorresteijn A The polychaete *Platynereis dumerilii *(Annelida): a laboratory animal with spiralian cleavage, lifelong segment proliferation and a mixed benthic/pelagic life cycle Bioessays 2004 26 314 325 10.1002/bies.10409 14988933

[B40] Liu N Han H Lasko P Vasa promotes *Drosophila *germline stem cell differentiation by activating *mei-P26 *translation by directly interacting with a (U)-rich motif in its 3' UTR Genes Dev 2009 23 2742 2752 10.1101/gad.1820709 19952109 PMC2788330

[B41] Seydoux G Strome S Launching the germline in *Caenorhabditis elegans*: regulaton of gene expression in early germ cells Development 1999 126 3275 3283 10393107 10.1242/dev.126.15.3275

[B42] Raz E Primordial germ-cell development: the zebrafish perspective Nat Rev Genet 2003 4 690 700 12951570 10.1038/nrg1154

[B43] Abascal F Zardoya R Posada D ProtTest: selection of best-fit models of protein evolution Bioinformatics 2005 21 2104 2105 10.1093/bioinformatics/bti263 15647292

[B44] Huelsenbeck JP Ronquist F Nielsen R Bayesian Analysis of Molecular Evolution Using MrBayes. Statistical Methods in Molecular Evolution 2005 New York: Springer

[B45] Guindon S Gascuel O A simple, fast, and accurate algorithm to estimate large phylogenies by maximum likelihood Syst Biol 2003 52 696 704 10.1080/10635150390235520 14530136

[B46] Rambaut A FigTree v1.3.1 http://tree.bio.ed.ac.uk/software/figtree/

[B47] Grassle J Grassle JF Sibling species in the marine pollution indicator *Capitella *(Polychaeta) Science 1976 192 567 569 10.1126/science.1257794 1257794

[B48] Seaver EC Paulson DA Irvine SQ Martindale MQ The spatial and temporal expression of *Ch-en*, the engrailed gene in the polychaete *Chaetopterus*, does not support a role in body axis segmentation Dev Biol 2001 236 195 209 10.1006/dbio.2001.0309 11456454

[B49] Seaver ED Kaneshige LM Expression of 'segmentation' genes during larval and juvenile development in the polychaetes *Capitella *sp. I and *H. elegans* Dev Biol 2006 289 179 194 10.1016/j.ydbio.2005.10.025 16330020

