# Supplementary material for: Somatic and germline expression of piwi during development and regeneration in the marine polychaete annelid Capitella teleta
Source: EvoDevo. 2011 May 5;2:10. doi: 10.1186/2041-9139-2-10 (PMC3113731; doi:10.1186/2041-9139-2-10)
Supplement: Additional file 1 — Accessions. This file includes a table of the GenBank and Swiss-Prot accession numbers used for sequence alignments and phylogenetic analysis. Also included are Joint Genome Institute protein identification numbers for sequences from the genomes of C. teleta, L. gigantea, and H. robusta. [file 2041-9139-2-10-S1.PDF]

Abbreviation in Figure	Species	GenBank Accession	Swiss-Prot Accession	JGI Protein ID
BmAGO3	Bombyx mori	NP_001098067.2		
BmAub	Bombyx mori	NP_001098066.2		
Amphi126411	Branchiostoma floridae	XP_002611937.1		
CePrg-1	Caenorhabditis elegans	NP_492121.1		
CePrg-2	Caenorhabditis elegans	NP_500994.1		
Ct-Piwi2	Capitella teleta			
Ct-Piwi1	Capitella teleta			
Ci-XP002130490	Ciona intestinalis	XP_002130490		
Ci-XP002120180	Ciona intestinalis	XP_002120180		
ChePiwi	Clytia hemisphaerica	ABY67112.1		
DmAub	Drosophila melanogaster	CAA64320.1		
DmPiwi	Drosophila melanogaster	NP_476875.1		
Hro-65566	Helobdella robusta			65566
Hro-75625	Helobdella robusta			75625
HsPiwi-l1	Homo sapiens	NP_004755.2		
HsPiwi-l3	Homo sapiens	NP_001008496.2		
HsPiwi-l4	Homo sapiens	NP_689644.2		
HsPiwi-l2	Homo sapiens	NP_060538.2		
Lg210915	Lottia gigantea			210915
Lg131825	Lottia gigantea			131825
MmPiwi-l1	Mus musculus	NP_067286.1		
MmPiwi-l4	Mus musculus	NP_808573.2		
MmPiwi-l2	Mus musculus	NP_067283.1		
Nv127599	Nematostella vectensis			127599
Nv79423	Nematostella vectensis			79423
Pdu-Piwi	Platynereis dumerilii	CAJ28986.1		
PcCniwi	Podocoryne carnea	AAS01181.1		
SpSeali	Strongylocentrotus purpuratus	NP_001107667.1		
SpSeawi	Strongylocentrotus purpuratus	AAG42533.1		
Ct-143595	Capitella teleta			143595
DmAgo-1	Drosophila melanogaster	NP_725341.1		
HsAgo-2	Homo sapiens		Q9UKV8.3	
HsAgo-1	Homo sapiens		Q9UL18.3	
